# Deadly Partners: Interdependence of Alcohol and Trauma in the Clinical Setting

**DOI:** 10.3390/ijerph6123097

**Published:** 2009-12-04

**Authors:** Amanda V. Hayman, Marie L. Crandall

**Affiliations:** 1 Department of Surgery, Northwestern University Feinberg School of Medicine, 251 East Huron St., Galter 3-150, Chicago, IL 60611-2950, USA; E-Mail: a-hayman@md.northwestern.edu; 2 Department of Surgery, Northwestern Unive rsity Feinberg School of Medicine, 676 N. St. Clair, Suite 650, Chicago, IL 60611, USA

**Keywords:** alcohol, trauma, intervention

## Abstract

Trauma is the leading cause of death for Americans aged 1 to 45. Over a third of all fatal motor vehicle collisions and nearly eighty percent of completed suicides involve alcohol. Alcohol can be both a cause of traumatic injury as well as a confounding factor in the diagnosis and treatment of the injured patient. Fortunately, brief interventions after alcohol-related traumatic events have been shown to decrease both trauma recidivism and long-term alcohol use. This review will address the epidemiology of alcohol-related trauma, the influence of alcohol on mortality and other outcomes, and the role of prevention in alcohol-related trauma, within the confines of the clinical setting.

## 1. Introduction

It has been well established that alcohol use increases the risk of a traumatic event, as well as overall poor health outcomes. An international study [1] concluded that alcohol contributes to 3.8% of all global deaths and 4.6% of all global disability-adjusted life years (DALYs). Alcohol is involved in 37% of all fatal motor vehicle collisions (MVCs) [2]. Further, risk of a MVC shows a dose-response with blood alcohol concentration (BAC), from five-fold at 80 mg/dl to 25-fold at 150 mg/dl [3]. Alcohol also plays a significant role in the 3.7 million non-fatal falls, 3.6 million non-fatal transport collisions (either motorized, bicycle, or pedestrian), and 60,000 non-fatal firearm injuries that occur annually in American adults 18–65 [4]. For example, alcohol use increases the risk of falling three to four times among young and middle-aged adults, according to a meta-analysis by Kool and colleagues [5]. According to the Institute for Research, Education, and Training in Addictions, over 20,000 (7.6 million per year) people enter emergency departments every day for alcohol-related injuries and illnesses [6].

## 2. Epidemiology

Heavy alcohol use was associated with nearly double the risk of violent injury among trauma patients presenting to an urban trauma center [7], and a Swiss study by Kuendig and colleagues [8] showed that nearly half of all emergency department (ED) patients who sustained non-fatal injuries report alcohol use prior to admission. This was consistent with all types of injuries and even low levels of alcohol consumption. Repeated studies confirm the very high prevalence of either acute or chronic alcohol use among trauma patients [9,10]. There is a very high unmet need for alcohol rehabilitation among these patients. Among a population of orofacial trauma victims who report any recent use of alcohol or drugs, 58% met criteria for alcohol abuse, although only a very small percentage report even receiving alcohol treatment. Penetrating trauma and male gender were two risk factors significantly associated with likelihood of testing positive for alcohol or illicit drugs at time of admission [11].

Patterns of alcohol use and abuse often begin early in life. A parental history of alcoholism is a very strong risk factor for problem drinking in offspring [12]. A survey conducted among over 43,000 members of the general American adult population queried participants about alcohol abuse and traumatic events. 34,653 were re-interviewed three years later. Results showed that the earlier the respondents reported drinking, the higher likelihood that they unintentionally injured themselves or someone else while drinking. More than a third of these events occurred in young adults (under age 25), despite this population only comprising 7% of those sampled [13]. Early interventions aimed at preventing alcohol use and abuse in the pediatric and adolescent population may prove to be an effective technique to decreasing alcohol-related traumatic events. However, young adults aren’t the only population at risk of alcohol-related trauma. Almost 10% of trauma patients over the age of 65 involve alcohol [14]. Alcohol was most highly associated with fall injuries in this population; therefore, clinicians should have a high suspicion of alcohol involvement in falls involving elderly patients.

## 3. Physiologic Outcomes

The role alcohol plays in trauma outcomes, including morbidity, mortality, and length of stay, remains controversial, both in human and animal studies. Multiple published studies report directly contradictory conclusions. The largest, and most recent study by Salim and colleagues [15], showed that mortality was significantly lower in moderate to severe traumatic brain injury (TBI) patients with a positive serum alcohol level on admission, although overall complications were higher. However, the retrospective nature of the study, as well as the absence of quantitative blood alcohol levels, calls in to question the statistic validity of these conclusions. Despite this, the authors suggested that, in the future, administering ethanol to TBI patients may be considered to improve mortality.

It has been more definitively established that alcohol does act as a confounder in clinical assessment. Intoxicated patients can present with a falsely depressed Glasgow Coma Scale (GCS), which may delay appropriate treatment, such as intubation or insertion of an intracranial pressure monitor. Golan and colleagues showed a 151 minute in delay in the insertion of such device in severely intoxicated patients (Blood alcohol level (BAL) ≥ 21.7 mmol/L) as compared to patients with negative BALs [16].

A study similar to Salim and colleagues’ [17] concluded that toxicology screens among TBI patients that were positive for methamphetamines or alcohol were associated with lower mortality, as well. However, when examining the effect of varying levels of serum alcohol among TBI patients, Shandro and colleagues found no difference in either short-term or long-term mortality [18]. Further, a prospective cohort study of TBI patients showed that higher BALs were associated with poorer performance on the Disability Rating Scale (DRS), but there was no association with short-term clinical outcomes or scores on the Functional Independence Measure (FIM) [19].

## 4. Psychological Outcomes

Further complicating the treatment of patients who experience alcohol-related trauma is the high rate of subsequent posttraumatic stress disorder (PTSD) and accompanying increase in alcohol use after the event. Patients with both alcohol dependence and PTSD have significantly worse physical and mental functioning than either affliction alone [20]. Therefore, not only does alcohol use increase the risk of experiencing a traumatic event, the converse is also true (*i.e.*, experiencing a traumatic event can increase the risk of subsequent alcohol use). McFarlane and colleagues found that patients who developed PTSD after a traumatic event had an increased risk of developing an incident alcohol use disorder [21]. Obviously, the compounded relationship between alcohol use begetting violence which begets heavier alcohol use complicates treatment for alcohol abuse after a traumatic event. It is important for practitioners to recognize the high prevalent co-morbidity of PTSD with alcohol abuse in patients who have experienced alcohol-related trauma and to focus treatment on both conditions.

## 5. Health Care Costs

Caring for intoxicated patients is more expensive than caring for non-intoxicated patients, especially in the trauma setting. This is partially explained by the increased number of required interventions and studies, given the unreliability of histories obtained from and physical examinations performed on these patients. In a 2009 study, O’Keefe and colleagues showed that intoxicated trauma patients were more likely to require invasive procedures (including intubation and urinary catheter insertion) and be admitted to either an inpatient unit or intensive care unit (ICU), when compared to non-intoxicated patients with similar clinical characteristics. His team calculated mean hospital charges were $1,833 greater per patient [22]. When this figure is multiplied by the millions of trauma patients seen in hospitals annually, the burden is substantial.

The Universal Policy Provision Law (UPPL) further complicates reimbursement for hospitals. This punitive law, which is only prohibited in thirteen U.S. states [23], allows insurance companies to deny coverage to individuals who have sustained alcohol-related healthcare charges. This places an unfair financial burden on trauma centers, as well as treating alcohol abuse as a crime, instead of a disease. This is compounded by the fact that alcohol abuse is more prevalent in the poor and correlates highly with mental illness and drug abuse, who are the most likely to have no insurance at all. More than 1% of the gross national product in high- and middle-income countries is attributable to the social and health costs of alcohol [1].

## 6. Prevention

By 2007, all 50 states had instituted a legal limit of 0.08% to be considered legally drunk, which was reduced from 0.1% starting in the late 1990s. This resulted in a statistically significant 5.2% reduction in single-vehicle-nighttime fatal traffic crashes in a before-and-after study of 19 jurisdictions [24]. Further, ten states have instituted “zero tolerance” laws for drivers under 21 years of age. The remainder of states uses 0.01 or 0.02% as the legal limit for drivers under 21, in compliance with the National Highway Systems Designation Act of 1995, which is required in order to receive federal highway funds [2]. An analysis of the effect of the zero tolerance law, as well as other underage drinking laws such as purchase and possession, suggests that 732 lives per year are saved as a result of its implementation, which gives support to the argument for all states to institute similar zero tolerance laws [25]. A similar study by the same group confirmed that legislation aimed at curbing drunk driving was effective in reducing fatal MVCs among adults, as well.

Another intriguing technique for preventing alcohol-related violence is increasing the price of alcohol. The strong relationship between violence and alcohol use has been repeatedly established [7], so one could hypothesize that increasing the price of alcohol results in a decrease in alcohol consumption and, subsequently, violent injury. A similar premise has been proven in studies that demonstrated that increasing cigarette prices decreases youth smoking [26]. In fact, multiple studies discussed by Jonathan Sheperd in his article discussing public health interventions aimed at decreasing alcohol-related violence [27] showed an inverse relationship between acts of violence (specifically child and intimate partner abuse) and alcohol prices [28,29]. Hindering access to alcohol, either by increasing taxes or restricting retail sales, could provide an effective technique for preventing alcohol-related traumatic events.

## 7. Interventions

Hospital admission after a traumatic event while intoxicated offers an excellent opportunity for therapeutic interventions aimed at rehabilitation. In 2005, The American College of Surgeons’ Committee on Trauma (ACSCOT) issued the SBI (screening and brief intervention) mandate that required that all Level I trauma centers systematically screen for problem drinkers and provide brief interventions for those that screen positive [6]. Work by Gentilello and colleagues suggested that an SBI performed in an inpatient setting could potentially result in long-term decrease in alcohol intake by the injured patient [30].

Unfortunately, not all studies have shown that interventions by health care providers are effective. One study by Roudsari and colleagues did not detect any decrease in repeat injuries, either alcohol-related or otherwise, six and twelve months after the administration for a brief alcohol intervention to trauma patients as compared to patients who did not receive the intervention [31]. However, one innovative study involved active Alcoholics Anonymous members visiting patients after alcohol-related trauma that required admission to the hospital. This 30- to 60-minute visit resulted in a statistically significant increase in abstinence from alcohol up to six months after discharge, as well as for initiation of treatment or self-help. This approach is especially enticing since it involves individuals outside of the treatment team, which could be seen as more approachable and empathetic to the trauma victim. Further, this type of community outreach fulfills the twelfth step of the AA program for alcoholics and does not add to health care costs or time burdens on the care team [32].

One barrier to proper treatment is identifying which patients are at the greatest risk of repeat injury. A study of all level I trauma centers in the U.S. reported only a 25% screening rate of patients who were deemed to be problem drinkers, despite the American College of Surgeons (ACS) alcohol screening and brief intervention mandate [33]. A study at Los Angeles County Hospital examined a single item binge drinking screen that was 76% sensitive in identifying patients who met criteria for alcohol abuse. Risk factors for alcohol abuse were male gender and substance abuse at the time of injury [34]. This simple screen could more efficiently identify those who would benefit from assessments or interventions with the hopes of decreasing trauma recidivism.

Given the conflicting evidence regarding the effectiveness of in-hospital interventions aimed at encouraging abstinence in trauma patients, clinicians may be reluctant to invest time and thus cost in performing these interventions. However, a cost-benefit analysis performed by Gentilello and colleagues confirmed that a brief intervention results in a net cost savings of 89 USD per patient screened and 330 USD for each patient offered an intervention, which is estimated as 1.82 billion USD annually [35].

## 8. Conclusions

Alcohol use not only doubles the risk of being involved in a traumatic event, both penetrating and blunt, but it also can complicate the initial evaluation and result in higher health care costs per traumatic event. The interdependence between subsequent development of PTSD and either incident or prevalent alcohol abuse further increases the complexity and cost of caring for this population after the event. Although the success of in-hospital interventions has been mixed, given the relative low cost associated with brief interventions by either clinicians or peers, the benefits likely outweigh the costs. A number of tools have been developed that have been helpful at identifying problem drinking and targeting which patients may benefit from such an intervention. However, perhaps the greatest impact in reducing alcohol-related trauma is made via preventative efforts aimed at children and adolescents, and especially via legislation regarding speeding and blood alcohol level limits, aimed at both adult and underage drivers.
